# Development and Usability Evaluation of an Art and Narrative-Based Knowledge Translation Tool for Parents With a Child With Pediatric Chronic Pain: Multi-Method Study

**DOI:** 10.2196/jmir.8877

**Published:** 2017-12-14

**Authors:** Kathy Reid, Lisa Hartling, Samina Ali, Anne Le, Allison Norris, Shannon D Scott

**Affiliations:** ^1^ Faculty of Nursing University of Alberta Edmonton, AB Canada; ^2^ Stollery Children's Hospital Alberta Health Services Edmonton, AB Canada; ^3^ Department of Pediatrics University of Alberta Edmonton, AB Canada; ^4^ Women & Children's Health Research Institute University of Alberta Edmonton, AB Canada

**Keywords:** pain, child health, parents, art

## Abstract

**Background:**

Chronic pain in childhood is increasingly being recognized as a significant clinical problem for children and their families. Previous research has identified that families want information about the causes of their child’s chronic pain, treatment options, and effective strategies to help their child cope with the pain. Unfortunately, parents have reported that finding this information can be challenging.

**Objective:**

The aim of this study was to actively work together with children attending a pediatric chronic pain clinic and their parents to develop, refine, and evaluate the usability of an art and narrative-based electronic book (e-book) for pediatric chronic pain.

**Methods:**

A multiphase, multi-method research design employing patient engagement techniques was used to develop, refine, and evaluate the usability of an art and narrative based e-book for pediatric chronic pain management to facilitate knowledge translation for parents with a child with chronic pain. The multiple phases included the following: (1) qualitative interviews to compile parents’ narratives using qualitative interviews; (2) qualitative data analysis; (3) development of an e-book prototype; (4) expert clinician feedback; (5) parent usability evaluation, knowledge change, and confidence in knowledge responses using an electronic survey; (6) e-book refinement; and (7) dissemination of the e-book.

**Results:**

A 48-page e-book was developed to characterize the experiences of a family living with a child with chronic pain. The e-book was a composite narrative of the parent interviews and encompassed descriptions of the effects the condition has on each member of the family. This was merged with the best available research evidence on the day-to-day management of pediatric chronic pain. The e-book was vetted for clinical accuracy by expert pediatric pain clinicians. All parents that participated in the usability evaluation (N=14) agreed or strongly agreed the content of the e-book was easy to understand and stated that they would recommend the e-book to other families who have children with chronic pain. Our research identified up to a 21.4% increase in knowledge after using the e-book, and paired *t* tests demonstrated a statistically significant difference in confidence in answering two of the five knowledge questions (chronic pain is a disease involving changes in the nervous system; the use of ibuprofen is usually effective at controlling chronic pain); *t*_13_=0.165, *P*=.001 and *t*_13_=0.336, *P*=.002, respectively, after being exposed to the e-book.

**Conclusions:**

Our results demonstrate that parents positively rated an e-book developed for parents with a child with chronic pain. Our results also identify that overall, parents’ knowledge increased after using the e-book, and confidence in their knowledge about chronic pain and its management increased in two aspects after e-book exposure. These results suggest that art and narrative-based knowledge translation interventions may be useful in transferring complex health information to parents.

## Introduction

Chronic pain (recurrent or persistent pain lasting longer than 3-6 months) in childhood is increasingly being recognized as a significant problem, affecting between 15% to 39% of children and their families [1-4]. Despite the high incidence of chronic pain in children, it is often underrecognized and undertreated by clinicians [5], and there are very few specialized pediatric pain clinics, resulting in complex and lengthy referral processes. Headache is the most common chronic pain complaint expressed by children, followed by abdominal and musculoskeletal pain [6]. Children with chronic pain may experience increased vulnerability to pain, depression, and anxiety, as well as decreased confidence in the ability to function [7]. Parents of children with chronic pain report increased personal levels of depression, anxiety, and distress [8]. The priority of pediatric chronic pain treatment is to return the child to a functional state where they are engaging in daily activities. Three types of interventions comprise pediatric chronic pain treatment: pharmacological, physical, and psychological interventions. Treatment must also address education and support of families’ reactions to their child’s pain and how they best support their child [9]. Previous research has identified that families want information about the causes of their child’s chronic pain and various treatment options, as well as effective strategies to help their child cope with the pain [10]. Specifically, this research surveyed parents (N=14) about expectations before attending the pain clinic and identified that having information about the causes of their child’s chronic pain was important or very important to 93% (13/14) of respondents and that having information about medications for pain was important or very important to 72% (10/14); 86% (12/14) of the parents rated reading materials as important. Unfortunately, finding this information has been reported to be challenging for parents [10].

Failure to implement the best available research is pervasive in child health [11], even for common pediatric conditions such as pain, fever, and asthma [12-15]. To date, knowledge translation (KT) efforts have largely focused on ensuring that health care professionals use the latest research to inform their practice; however, emerging evidence suggests that initiatives that target health care consumers (eg, parents) can inform their decision making and shape their treatment expectations [16,17]. KT in child health is unique given the family-centered approach to care and the often essential extent of parental involvement in the care of children. Research has demonstrated that strategies to increase KT to parents by pediatric health care professionals can reduce health care utilization and improve outcomes [18-23]. The power of focusing on KT for both children and parents has yet to be fully realized.

There is an unprecedented demand for consumer-friendly, reliable, evidence-based health information for patients and their families because of the complex nature of health care, the rapid increase in the amount of health research, and the increased accessibility to research offered through the Internet [24,25]. Innovative mediums are superior to traditional standard health sheets for transferring information to consumers [26,27]. Thus, the use of art and narrative-based approaches holds promise for effectively transferring research evidence to patients and families [28]. Previous research in this area illustrates the power of art and narrative-based forms to communicate, engage with, and influence individuals [25,29-34]. To date, limited research has explored using art and narrative forms on digital platforms. The purpose of this research was to actively work together with children attending the pediatric chronic pain clinic in our children’s hospital and their parents to develop, refine, and evaluate the usability of an art and narrative-based e-book for pediatric chronic pain.

## Methods

A seven-phase, multi-method design employing patient engagement techniques was used to develop, refine, and evaluate the usability of an art and narrative-based e-book for pediatric chronic pain management to facilitate knowledge translation. Ethics approval was received from our institutional ethics board.

### Compilation of Knowledge User Narratives (Intervention Development)

The project coordinator (trained in qualitative methods and supervised by the principal investigator [PI]) conducted semistructured qualitative interviews with a purposeful sample of family members with a child with chronic pain from the Stollery Children’s Hospital (Edmonton, Alberta, Canada). Semistructured interviews ensured the acquisition of rich description and data while simultaneously allowing children and parents the freedom to respond and illustrate their experiences with pediatric chronic pain [35]. Questions focused exclusively on the experience of having a child with chronic pain and moved from general to specific, with interviews later in the data collection period becoming increasingly focused. Themes that were explored in the interviews included the effects of having a child with chronic pain on family life, social life, vacations, and work schedules; feelings regarding the diagnosis; expenses associated with having a child with chronic pain; experiences with the chronic pain clinic and health professionals involved; and parents’ hopes or wishes. The interviews were digitally captured and cleaned before the coordinator coded the interviews.

### Qualitative Data Analysis

Data collection and analysis occurred iteratively [35,36]. As analysis progressed, the interviews became more precise and purposeful [37]. Data collection continued until saturation of major categories [38] was achieved. Analysis occurred in three phases: coding, categorizing, and developing themes. Coding of the interview transcripts was led by the project coordinator and supervised by the PI (SS). Similar codes were then grouped into categories, and larger themes with memos were used to capture the analytic process. NVIVO 11 (QSR International Pty Ltd.) version 11, 2015 was used to facilitate data management during the analytic phase. Trustworthiness of the findings was ensured through iterative data collection and analysis. A summary of the interview themes (effect on the family, emotional experiences with chronic pain, experiences with the chronic pain clinic, families’ information needs, and families’ hopes and needs) with accompanying data excerpts from the interviews was shared with a creative writer who worked with the research team to develop the narrative for the e-book.

### Developing Prototypes (Intervention Development)

The development of the e-book prototype involved the creation of the composite narrative (compilation of common themes from the interviews), integrating the best available research on pediatric chronic pain management into the composite narratives, development of the artwork, and graphic display of the narrative and artwork. Our team worked with a creative writer, illustrator, graphic designer, and videographer to develop the e-book. We embedded information icons throughout the e-book to provide the best available research evidence on key pediatric chronic pain principles, as well as clear language explanations. Furthermore, additional resources (ie, breathing exercises) were also embedded to help children and families cope with managing chronic pain.

### Expert Clinician Feedback

After the e-book prototype was developed, it was shared with a multidisciplinary team of clinician experts at the pediatric chronic pain clinic that included a physician, psychologist, and nurse practitioner. The pediatric chronic pain service at the Stollery Children’s Hospital (Edmonton, Alberta, Canada) provides treatment and care for children 17 years and below who experience chronic, difficult to manage pain. The clinicians reviewed the e-book for knowledge accuracy and are ideally suited for this assessment as they are well abreast of the best available research evidence in this highly specialized field.

### Prototype Usability Evaluation and Knowledge Change

A link to prototypes of the e-book, usability test, and pre and post knowledge tests were emailed (by clinic staff) to all 25 parents with a valid email address in the Stollery chronic pain clinic [39,40] database. Consent was implied if the Web-based survey was completed and submitted. Parents were asked to complete a Web-based survey that assessed their perceptions of the prototype using a 5-point Likert scale and included elements informed by a systematic search of over 180 usability evaluations [41]: (1) usability, (2) aesthetics, (3) language, (4) level of engagement, (5) ease-of-use, (6) knowledge provided, (7) preference of form over traditional dissemination venues, (8) value-added (please see questions in Multimedia Appendix 1). Furthermore, parents had the opportunity to provide free text feedback on areas that required revisions or more information. To evaluate parents’ knowledge of pain, parents were asked to answer five true or false knowledge questions about pediatric chronic pain and to rate their level of confidence in their responses before viewing the e-book. Evaluation of pain knowledge focused on five main topics: (1) what is chronic pain, (2) what is acute pain, (3) effects of chronic pain on children, (4) how to treat chronic pain, and (5) ibuprofen use and chronic pain. Parents rated their level of confidence in their response to each question using a 5-point Likert scale (very sure to very unsure). After completing the baseline knowledge test, the parents were to read the e-book, and knowledge questions were answered again to assess short-term knowledge changes. Participants were again asked to rate their confidence.

The data was cleaned and managed according to industry standards. Data was entered into Statistical Package for the Social Sciences (SPSS) version 21 (IBM Corp), and descriptive statistics (eg, frequencies), measures of central tendency, as well as paired *t* tests were completed. To uncover potential usability issues, the free text data was analyzed using content analysis, and any suggestions for revision were grouped and examined [42].

### Prototype Refinement

On the basis of the results from the clinician experts and the parent usability evaluation, changes were made to the e-book.

### Dissemination

The finalized e-book was disseminated through the Stollery Children’s Hospital via the chronic pain team and the Pain 101 course for parents (a clinic specific course for children and parents who are cared for by the pediatric chronic pain team) through established social media platforms including investigators’ Web pages (www.echo.ualberta.ca) and TREKK (www.trekk.ca), a network of health professionals and parents whose goals are to improve emergency care for children. Each of these Web pages is enabled with Google Analytics, thereby allowing investigators to track the visitor behavior attributes such as the number of visits to the e-tools, number of page views, and the average length of time viewing each e-tool [36]. A PDF version of the book was also shared on the local Alberta Health Services website and added to the chronic pain toolkit on the Canadian Association of Paediatric Health Centres Web page. After usability testing was completed on the e-book version, a hard copy of the book was published and distributed to junior high and high schools in and around the Edmonton area, local libraries, families seeking care through the pediatric pain clinic, and the two major children’s hospitals in Alberta. An audiobook was generated to serve as an accompaniment for the e-book or hardcopy book.

## Results

### The Product

**Figure 1:**
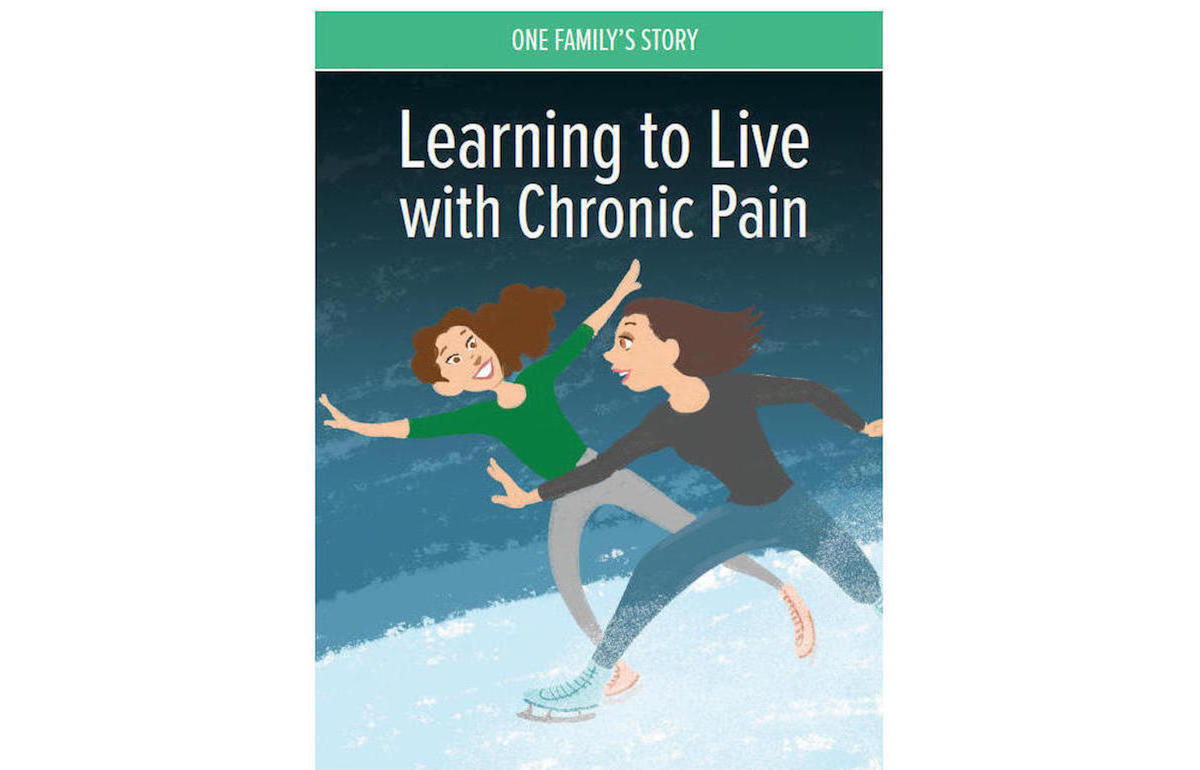
Cover of the electronic book (e-book).

**Figure 2:**
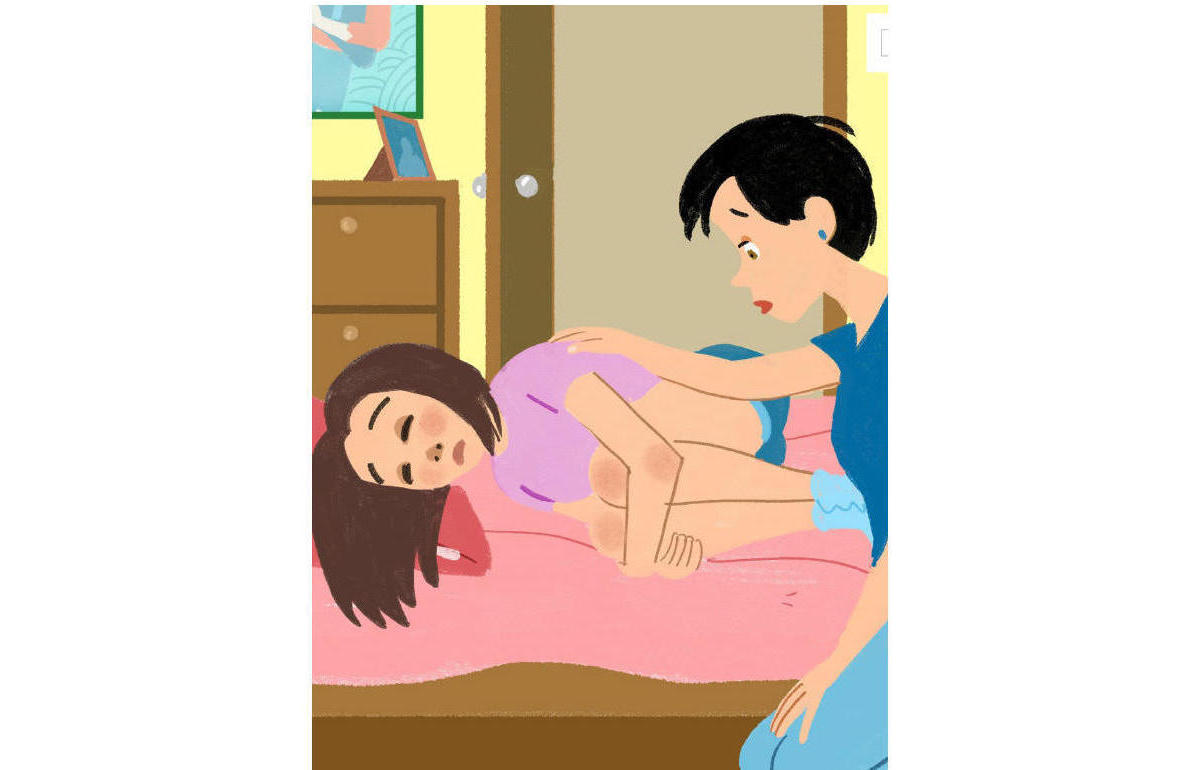
Illustration from the electronic book (e-book).

**Figure 3:**
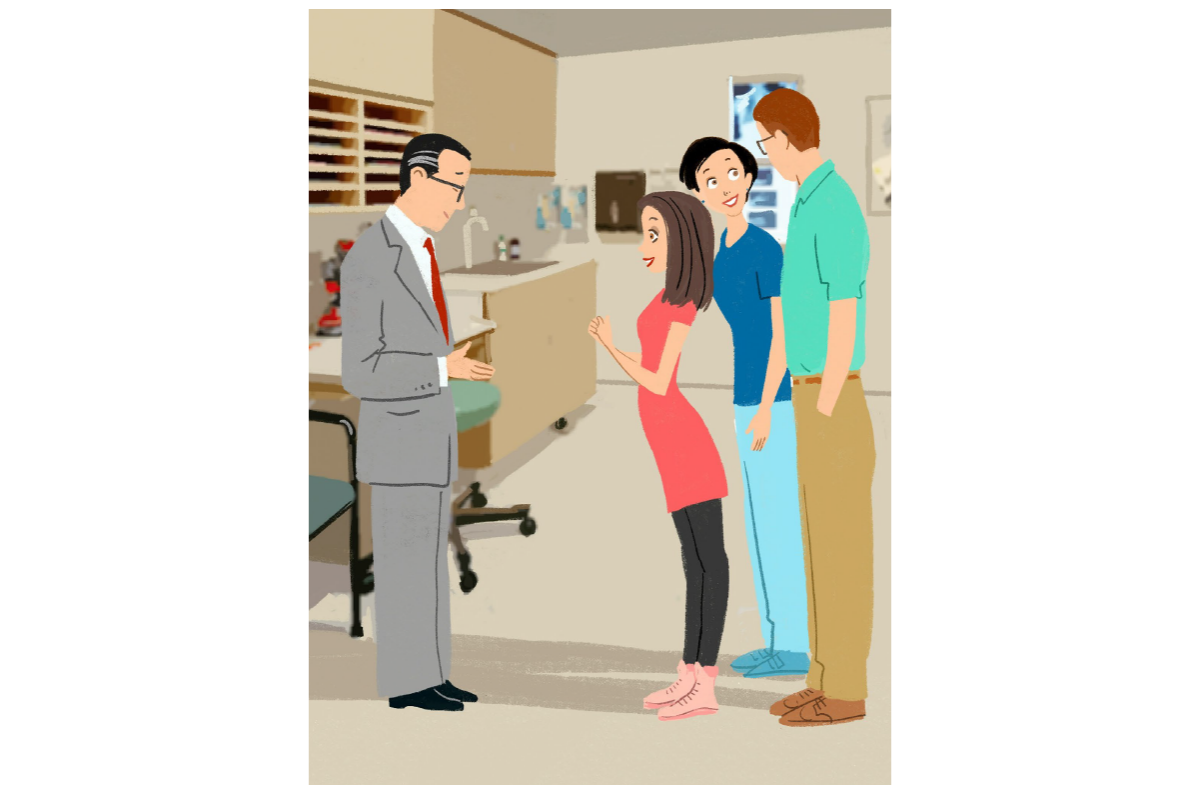
Illustration from the electronic book (e-book).

**Figure 4:**
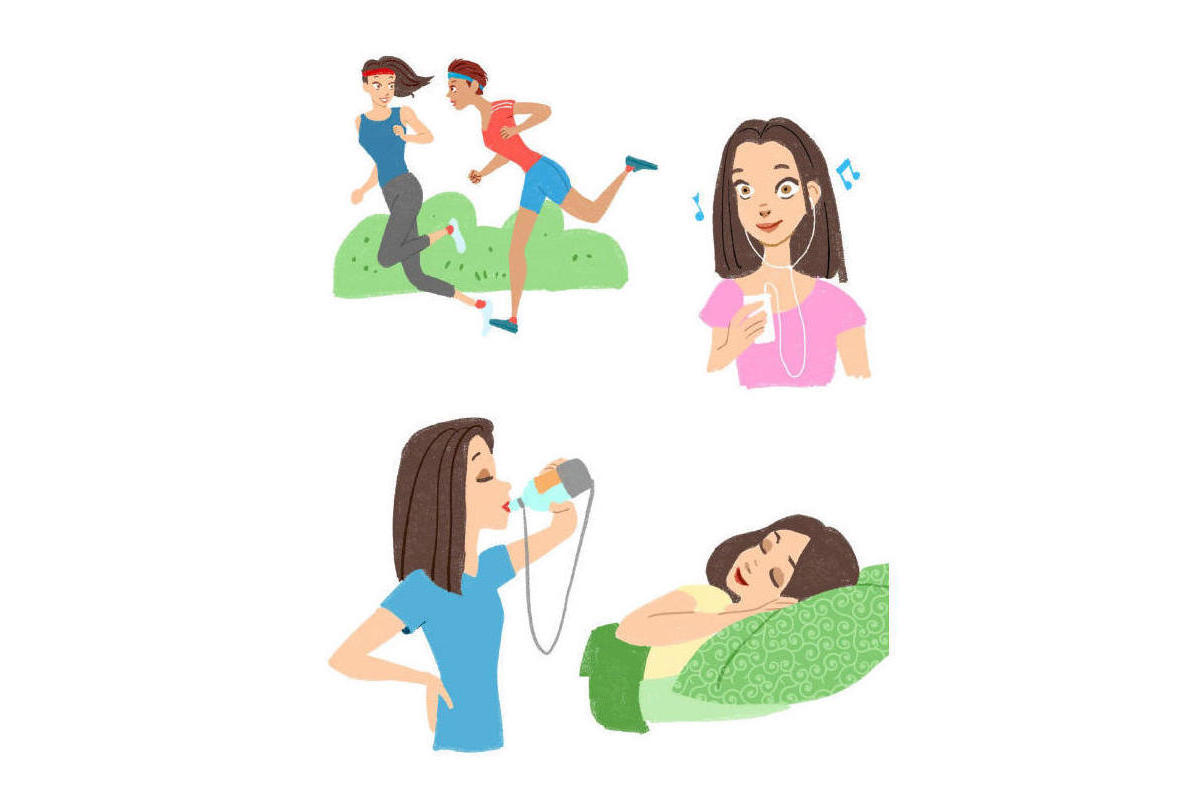
Illustration from the electronic book (e-book) depicting four different pain management strategies.

The creative writer generated a story based on interviews with parents or caregivers of 17 children presenting to the chronic pain clinic. The 48-page book was written in the perspective of a mother and was designed to characterize the experiences of a family living with a child with chronic pain (Figure 1). The e-book encompassed descriptions of the effects the condition plays on each member of the family, including the mother, father, siblings, and the child living with chronic pain. The story highlighted the difficulties and struggles of finding resources to improve the quality of life of a child with chronic pain (Figure 2). To ensure readers understood certain terms, definitions were provided in the index at the end of the e-book. This is further complemented by a series of exercise suggestions that families struggling with chronic pain may use (Figures 3 and 4).

### Usability Testing Results

Evaluation of the chronic pain e-book included assessments of usability and pretest and posttests of pain knowledge, as well as confidence in knowledge responses. Emails were sent to 25 parents from the Stollery chronic pain clinic (all parents with valid email addresses in the clinic database); 22 surveys were started and 14 were fully completed. All 14 surveys submitted were completed by females in the household; 13 of the individuals were mothers, and one was a grandmother. Eleven participants completed or had taken some form of postsecondary education, one completed high school, and one had some high school. Eleven participants indicated that their family or child had been dealing with the chronic pain for 2 or more years, two families’ participants indicated that the family had been struggling with the chronic pain for 1 to 2 years, and one participant stated that the family had been struggling with chronic pain for 6 to 12 months. Table 1 describes the demographics of the study population.

**Figure 5:**
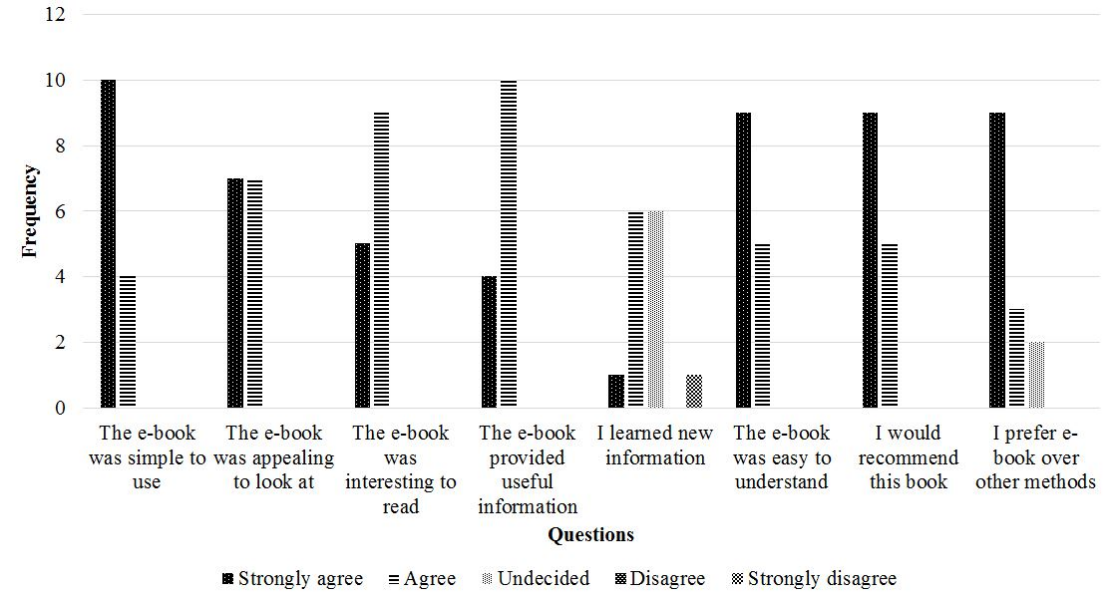
Frequency of participant answers on usability testing questionnaire.

Parental reaction to the e-book was generally positive (Figure 5). Usability testing indicated that, in general, parents all agreed or strongly agreed that the e-book was simple to use. Similarly, all parents felt that the book was appealing to look at and that it was interesting to read. In terms of usefulness, again, all parents strongly agreed or agreed that the information provided in the e-book would be useful for families struggling with a child’s chronic pain. When asked if parents felt that they had learned more information from the e-book, 1 parent strongly disagreed, 6 parents were undecided, 6 agreed, and 1 parent strongly agreed.

All 14 parents agreed or strongly agreed that the content of the e-book was easy to understand, and similarly, all parents stated that they would recommend the e-book to other families who have children with chronic pain. When asked if parents preferred the e-book (composite parent narrative with evidence-based information supplemented with illustrations) over standard ways (text only, ie, waiting room pamphlets, discharge instructions, etc.) to receive health information, 2 parents stated that they were undecided, and 12 parents agreed or strongly agreed. Comments on the usability testing survey indicated that parents found that the book paralleled aspects of their own lives. For instance, some comments included:

This eBook is essentially our lives. It was nice to read a story that we could relate to so easily.

I liked the book but I shouldn’t have done the survey at work as it made me cry remembering our own situation with our daughter.

The book provides an accurate account of what it is like living in a family when a child lives with chronic pain.

One parent noted that the outcomes to the solutions provided in the e-book may not be similar across all families:

From my perspective, it showed the Pain Clinic as being the rosy solution to life of chronic pain. It paints the picture that this will be the solution. For some families, it may well be, but for others, it will be a tool to give them knowledge and understanding but it won’t be the cure. It was mentioned lightly there is no cure for chronic pain, but families need to understand this going in.

One parent felt the book was a great resource for knowledge dissemination:

The eBook was great. Hopefully it will aid in getting the word out there to parents who are struggling to help their kids suffering from chronic pain; that there is help.

### Knowledge Evaluation and Response Confidence

Across the five knowledge topics, we found up to a 21.4% increase in knowledge (range 0-21.4%; Table 2) after being exposed to the e-book. In one of the knowledge topics (topic 4) before reading the e-book, 100% of the participants successfully identified the correct response, and this knowledge was retained post exposure. In three topics, knowledge gains after exposure to the e-book were 7.1% (topic 5), 14.3% (topic 1), and 21.4% (topic 2). In topic 3, there was a knowledge loss of 7.1% after reading the book. We did find a statistically significant difference in confidence of parent answers for two of the five topics (Table 3). Paired *t* tests showed that there was a statistically significant difference in confidence in answering the true or false questions “chronic pain is a disease involving changes in the nervous system” and “the use of ibuprofen is usually effective at controlling chronic pain.”

**Table 1:** Survey participant demographic characteristics (N=14).

Variable		n (%)
**Sex**		
	Female	14 (100.0)
	Male	0 (0.0)
**Age (years)**		
	31-40	2 (14.3)
	41-50	9 (64.3)
	≥51	3 (21.4)
**Marital status**		
	Married	11 (78.6)
	Single	3 (21.4)
**Education**		
	Some high school	1 (7.1)
	High school diploma	1 (7.1)
	Some postsecondary	2 (14.3)
	Postsecondary certificate or diploma	9 (64.3)
	Graduate degree	1 (7.1)
**Relationship to child**		
	Parent	13 (92.9)
	Grandparent	1 (7.1)
**Age of child (years)**		
	13	3 (21.4)
	16	4 (28.6)
	17	5 (35.7)
	18	1 (7.1)
	Missing	1 (7.1)
**Length of pain**		
	6-12 months	1 (7.1)
	1-2 years	2 (14.3)
	≥2 years	11 (78.6)
**Type of pain (parents select all that apply)**		
	Headache	8 (57.1)
	Abdominal	7 (50.0)
	Muscular	6 (42.9)
	Arthritis	3 (21.4)
	Complex regional pain syndrome	4 (28.6)
	Other	5 (35.7)
**Electronics used at home (parents selected all that applied)**		
	Desktop computer	4 (28.6)
	Laptop	11 (78.6)
	Tablet	12 (85.7)
	Mobile phone	13 (92.9)
	E-reader	4 (28.6)

**Table 2:** Changes in knowledge and response confidence before and after exposure to the electronic book (e-book).

Topic or question (correct response)	Scenario		Frequency, n (%)
	Pre	Post	
Chronic pain is a disease involving changes in the nervous system. (true)	T^a^	F^b^	0 (0.0)
	F	T	2 (14.3)
	T	T	12 (85.7)
	F	F	0 (0.0)
Acute pain is a short-term pain caused by disease or injury. (true)	T	F	0 (0.0)
	F	T	3 (21.4)
	T	T	11 (78.6)
	F	F	0 (0.0)
Children with chronic pain often have a difficult time doing regular activities (eg, going to school and hobbies). (true)	T	F	1 (7.1)
	F	T	0 (0.0)
	T	T	13 (92.9)
	F	F	0 (0.0)
Using more than one type of treatment (eg, counseling and medications) at the same time can be more effective at controlling chronic pain than individual treatments. (true)	T	F	0 (0.0)
	F	T	0 (0.0)
	T	T	14 (100.0)
	F	F	0 (0.0)
The use of ibuprofen is usually effective at controlling chronic pain. (false)	T	F	1 (7.1)
	F	T	0 (0.0)
	T	T	0 (0.0)
	F	F	13 (92.9)

^a^T: true.

^b^F: false.

**Table 3:** Changes in response confidence before and after exposure to the electronic book (e-book).

Topic or question (correct response)	Pre/Post-test	Confidence					*P* value
		Very unsure n (%)	A little unsure n (%)	Neither sure or unsure n (%)	A little sure n (%)	Very sure n (%)	
Chronic pain is a disease involving changes in the nervous system. (true)	Pre	2 (14.3)	6 (42.9)	0 (0.0)	3 (21.4)	3 (21.4)	.001^a^
	Post	0 (0.0)	0 (0.0)	0 (0.0)	3 (21.4)	11 (78.6)	
Acute pain is a short-term pain caused by disease or injury. (true)	Pre	0 (0.0)	1 (7.1)	1 (7.1)	3 (21.4)	9 (64.3)	.17
	Post	0 (0.0)	0 (0.0)	0 (0.0)	3 (21.4)	11 (78.6)	
Children with chronic pain often have a difficult time doing regular activities (eg, going to school and hobbies). (true)	Pre	0 (0.0)	0 (0.0)	0 (0.0)	2 (14.3)	12 (85.7)	.34
	Post	0 (0.0)	0 (0.0)	0 (0.0)	1 (7.1)	13 (92.9)	
Using more than one type of treatment (eg, counseling and medications) at the same time can be more effective at controlling chronic pain than individual treatments. (true)	Pre	0 (0.0)	0 (0.0)	1 (7.1)	1 (7.1)	12 (85.7)	>.99
	Post	0 (0.0)	0 (0.0)	1 (7.1)	1 (7.1)	12 (85.7)	
The use of ibuprofen is usually effective at controlling chronic pain. (false)	Pre	0 (0.0)	2 (14.3)	3 (21.4)	4 (28.6)	5 (35.7)	.002^a^
	Post	0 (0.0)	0 (0.0)	0 (0.0)	1 (7.1)	13 (92.9)	

^a^Significant at *P*<.05.

## Discussion

### Principal Findings

This study evaluated the usability of a novel knowledge translation tool for parents of children with chronic pain. Parental results on the usability on the e-book were very positive, knowledge gains ranged from −7.1% to 21.4% after exposure to the e-book, and we identified that the e-book significantly increased parents’ confidence in two areas—(1) understanding that chronic pain is a disease involving the nervous system and (2) in the use of ibuprofen to treat or manage pain. Pain neuroscience education (PNE) is an important aspect of treatment for those who experience chronic pain that has been studied in adults; however, a recent review [43] of PNE for pediatric pain found no published studies that examine how educating parents impacts parental and child function in the context of their child’s pain. Our study is the first to examine how PNE, through the use of art and narrative, has significantly increased parental confidence in this area. Although there have been several books published by pediatric pain experts in recent years aimed at educating and helping parents manage their child’s chronic pain, none have completed an evaluation on the impact of their book on parental understanding of their child’s chronic pain [44-48].

Second, parental data from in our study highlighted the importance of *seeing their experience* in the e-book. This is not a unique phenomenon, and in fact, in some of our earlier research developing books for parents with a child with croup, we noted similar findings. In that study, parents reported that having elements of the narrative (story) mirror their experience validated their emotional reactions to their child’s condition and also provided assistance in managing the situation [31,32]. Furthermore, parents in our earlier research reported that employing a narrative with elements that mirrored their experience normalized the experience, and they felt reassured that other parents had experienced similar emotions. As a result, these parents stressed that this helped them feel less “alone,” and sharing similar experiences with other parents was important in decreasing their anxiety.

The notion of validation of experiences, identification, or mirroring of personal experience with the narrative, also called homophily, is fundamental to the success of stories [49] as a knowledge translation medium. Abrahamson [50] has identified that the narrative functions as an intellectual, cognitive, and emotional channel between the story and the reader. The mirroring between the narrative and personal experience facilitates engagement or “awakening” of the reader of the e-book to their previous experience [50] and applicability of information to real-life situations [31]. As a result, this parental validation confirms that the early phases of interviewing multiple parents to understand their experience and then working with a creative team to develop a composite narrative (bringing several parents’ experiences together in one story) that incorporates experiences from many parents is fundamental to the success of the e-book as a knowledge translation tool. Kreuter et al [49] further develops the fundamental role of personal identification with the narrative and stresses that stories “work by engaging an audience and modeling behaviors and their consequences.” Building on social cognitive theory, Kreuter and colleagues go further to add that the effect of modeling is increased with similarities in the narrative, particularly identification with story characters. In particular, the collection of interviews to obtain rich experiential data maybe particularly poignant when developing knowledge translation tools for chronic conditions such as pediatric chronic pain. Noel and colleagues stressed that the collection of pain narratives from parents with a child with chronic pain was able to capture the multidimensional nature of their experience [51].

Parents in our study identified that they preferred receiving health information in a narrative form rather than the standard information-based format (no story rather exclusive health information). Parents who evaluated the e-book (n=12) agreed or strongly agreed that they preferred the e-book over standard ways of receiving health information. Again, this finding mirrors our previous research [31,32] where parents preferred receiving health information in a story as compared with the standard medical information sheet. There are several potential reasons to explain this preference, including the ability of stories to promote engagement, stories aesthetic appeal, and their novelty. Oatley [52] offers another reason suggesting that human memory is predominantly story-based, and consequently, new information may be more easily integrated if it corresponds with an existing story component in memory. Schrank et al [53] expand on Oatley’s notion and outline that indexing of new information in human memory (for future reuse) is increased when links can be made between personal experience and the narrative (e-book). These links facilitate assimilation of new health information with existing memory and increase the potential for integration and use of the new information in future situations [31]. The work of Hinyard and Kreuter reinforce this notion and add that whereas there is only an emerging evidence base on using narrative to transfer health information, it is apparent that narrative information is processed differently than nonnarrative information.

Related research also demonstrates that not only do consumers prefer a narrative format, this narrative format can also spur clinically relevant improvements to patient outcomes. For instance, Houston et al [24] tested the effectiveness of cultural appropriate storytelling to improve blood pressure in 230 African Americans with hypertension. They discovered that the patients with baseline uncontrolled blood pressure that received the narrative-based intervention had substantial and significant improvements in blood pressure at 6 months. Although the evidence base on the use of narrative to influence behavior change and clinical outcomes is emerging, it is apparent that there is potential for this modality, given its obvious applicability with conventional human communication and connections.

### Conclusions

Our research holds promise for future development, application, and effectiveness testing of art and narrative-based knowledge translation interventions for transferring complex health information to parents. Our results demonstrated that parents positively rated an e-book developed for parents with a child with chronic pain and that they preferred this format over standard information-based approaches to transferring health information. Our results identified that parents experienced −7.1% to 21.4% increases in knowledge after exposure to our e-book and identified that parents’ confidence in their chronic pain management knowledge increased in two aspects after exposure to our art and narrative-based e-book. These novel findings highlight the potential for digital art and narrative-based knowledge translation tools given their congruence with human communication and learning approaches. Our findings suggest that future research employing digital art and narrative-based tools for knowledge transfer is needed and worthwhile, in particular, assessing these approaches with different types of clinical conditions (ie, acute vs chronic health conditions) and different types of parents (ie, demographics, educational levels, ethnic backgrounds, and learning styles).

## Appendix

Multimedia Appendix 1 Webpage access to the electronic book (e-book).

## Abbreviations

e-book: electronic book
KT: knowledge translation
PI: principal investigator
PNE: pain neuroscience education
