# Supplementary material for: Development and Usability Evaluation of an Art and Narrative-Based Knowledge Translation Tool for Parents With a Child With Pediatric Chronic Pain: Multi-Method Study
Source: J Med Internet Res. 2017 Dec 14;19(12):e412. doi: 10.2196/jmir.8877 (PMC5746621; doi:10.2196/jmir.8877)
Supplement: Multimedia Appendix 1 [file jmir_v19i12e412_app1.pdf]

Learning to Live with Chronic Pain

TREKK: http://trekk.ca/teams/chronic-pain-ebook-audio

Alberta Health Services website: https://www.albertahealthservices.ca/stollery/Page14314.aspx
